# Effect of a vitamin/mineral supplement on children and adults with autism

**DOI:** 10.1186/1471-2431-11-111

**Published:** 2011-12-12

**Authors:** James B Adams, Tapan Audhya, Sharon McDonough-Means, Robert A Rubin, David Quig, Elizabeth Geis, Eva Gehn, Melissa Loresto, Jessica Mitchell, Sharon Atwood, Suzanne Barnhouse, Wondra Lee

**Affiliations:** 1 Autism/Asperger's Research Program, Arizona State University, Tempe, AZ, USA; 2 Health Diagnostics and Research Institute, South Amboy, NJ, USA; 3 Integrative Developmental Pediatrics, Tucson AZ, USA; 4 Department of Mathematics, Whittier College, Whittier, CA, USA; 5 Doctor's Data, St. Charles, IL, USA; 6 Southwest College of Naturopathic Medicine, Tempe, AZ, USA

## Abstract

**Background:**

Vitamin/mineral supplements are among the most commonly used treatments for autism, but the research on their use for treating autism has been limited.

**Method:**

This study is a randomized, double-blind, placebo-controlled three month vitamin/mineral treatment study. The study involved 141 children and adults with autism, and pre and post symptoms of autism were assessed. None of the participants had taken a vitamin/mineral supplement in the two months prior to the start of the study. For a subset of the participants (53 children ages 5-16) pre and post measurements of nutritional and metabolic status were also conducted.

**Results:**

The vitamin/mineral supplement was generally well-tolerated, and individually titrated to optimum benefit. Levels of many vitamins, minerals, and biomarkers improved/increased showing good compliance and absorption. Statistically significant improvements in metabolic status were many including: total sulfate (+17%, p = 0.001), S-adenosylmethionine (SAM; +6%, p = 0.003), reduced glutathione (+17%, p = 0.0008), ratio of oxidized glutathione to reduced glutathione (GSSG:GSH; -27%, p = 0.002), nitrotyrosine (-29%, p = 0.004), ATP (+25%, p = 0.000001), NADH (+28%, p = 0.0002), and NADPH (+30%, p = 0.001). Most of these metabolic biomarkers improved to normal or near-normal levels.

The supplement group had significantly greater improvements than the placebo group on the Parental Global Impressions-Revised (PGI-R, Average Change, p = 0.008), and on the subscores for Hyperactivity (p = 0.003), Tantrumming (p = 0.009), Overall (p = 0.02), and Receptive Language (p = 0.03). For the other three assessment tools the difference between treatment group and placebo group was not statistically significant.

Regression analysis revealed that the degree of improvement on the Average Change of the PGI-R was strongly associated with several biomarkers (adj. R^2 ^= 0.61, p < 0.0005) with the initial levels of biotin and vitamin K being the most significant (p < 0.05); both biotin and vitamin K are made by beneficial intestinal flora.

**Conclusions:**

Oral vitamin/mineral supplementation is beneficial in improving the nutritional and metabolic status of children with autism, including improvements in methylation, glutathione, oxidative stress, sulfation, ATP, NADH, and NADPH. The supplement group had significantly greater improvements than did the placebo group on the PGI-R Average Change. This suggests that a vitamin/mineral supplement is a reasonable adjunct therapy to consider for most children and adults with autism.

**Trial Registration:**

**Clinical Trial Registration Number: **NCT01225198

## Background

Vitamins and minerals (elements) are, by definition, essential for human health, primarily due to their critical function as enzymatic cofactors for numerous reactions in the body, such as the production of neurotransmitters and fatty acid metabolism Historically attention has focused on inadequate intake of vitamins and minerals due to poor diet as a major contributing factor to many child health problems in the US and around the world, including anemia (low iron), hypothyroid (low iodine), scurvy (vitamin C deficiency), and rickets (calcium and/or vitamin D deficiency). More recently the focus has shifted to the relationship between relative metabolic disturbances and developmental disorders, for example those associated with attention deficit disorder [1-5], learning disorders [6], and intellectual development [7]. Children with autism sometimes have limited, self-restricted diets, and in this paper we further investigate the hypothesis that nutritional insufficiency and metabolic imbalances may play a role in autism spectrum disorders (ASD) as well.

According to a recent survey of 539 physicians, vitamin/mineral supplements are among the most widely recommended medical interventions for autism, and are recommended by 49% of physicians for children with autism [8]. However, there have been relatively few treatment studies of vitamin/mineral supplements for children with autism. Three studies demonstrated that children with autism have impaired methylation (decreased SAM), decreased glutathione, and increased oxidative stress [9-11] compared to neurotypical children. Two open-label studies demonstrated that nutritional supplementation - with vitamin methyl-B12, folinic acid, and (in one of the studies) trimethylglycine - resulted in statistically significant improvements in methylation, glutathione, and oxidative stress [9,10]. A 30-week, double-blind, placebo-controlled study [12] of high-dose vitamin C (110 mg/ kg) found a reduction in autism severity as measured by the Ritvo-Freeman scale. There have been 11 double-blind, placebo-controlled studies of very high dose vitamin B6 with magnesium, with almost all showing positive behavioral improvements. However, the studies were somewhat limited by methodological problems including small sample size and the use of assessment tools of limited validity [13]. There was one published study of a multi-vitamin/mineral supplement for children with ASD [14], which used a randomized, double-blind, placebo-controlled design. None of the children in the study were on a vitamin/mineral supplement for two months prior to the study. They found that the treatment group generally improved more than the placebo group, with statistically significant greater improvements in sleep (p = 0.03) and gastrointestinal problems (p = 0.03), both of which are very common in autism [15-19].

Due to the promising results of the 2005 study of a moderate dosage multi-vitamin/mineral supplement [14], in 2007/2008 we conducted a small (n = 10) open-label pilot study of a customized vitamin/mineral supplement for children with ASD, which included extensive pre and post measurements of nutritional status (vitamins, minerals, amino acids) and metabolic functioning (oxidative stress, methylation, glutathione, sulfation, and neurotransmitters). The supplement was well-absorbed (as indicated by increases in blood levels and urinary excretion), and improved levels of glutathione and some neurotransmitters. The results of that pilot study were used to reformulate the supplement, adjusting the level of some ingredients slightly up or down based on the laboratory findings.

In 2008/2009 this revised "second generation" supplement was used to conduct a randomized, double-blind, placebo-controlled three-month treatment study, the results of which are being reported in this paper. As the preliminary phase to the treatment study, a detailed comparison study was conducted of the nutritional and metabolic status of the children with autism (N = 55, recruited for the treatment study) vs. neurotypical children of similar age, gender and locale. The significant findings of the baseline study [20] are summarized as follows. Levels of biomarkers for the neurotypical controls were in good agreement with accessed published reference ranges, which provided validation of the overall measurement methodology. The average levels of vitamins, minerals and most amino acids for the autism group were within published reference range for nutrients commonly measured in clinical care, but sometimes in the lower or higher end of the reference range. The autism group had many statistically significant differences (p < 0.001) in their average levels of biomarkers compared to the neurotypical group, including: Low levels of biotin, glutathione, S-adenosylmethionine (SAM), plasma adenosine-5'-triphosphate (ATP), reduced nicotinamide adenine dinucleotide (NADH), reduced nicotinamide adenine dinucleotide phosphate (NADPH), plasma sulfate (free and total), and plasma tryptophan; also high levels of oxidative stress biomarkers and evidence of impaired methylation (high uridine). A stepwise, multiple linear regression analysis demonstrated significant associations between all three autism severity scales and several groups of biomarkers, including vitamins (adjusted R^2 ^of 0.25-0.57), minerals (adj. R^2 ^of 0.22-0.38), and plasma amino acids (adj. R^2 ^of 0.22-0.39). Thus, it appears that many of these biomarkers are different in children with autism and significantly associated with variations in autism severity. These results then lay the foundation and provide the rationale for the present treatment study.

This paper presents the effect of the revised "second generation" supplement on the nutritional/metabolic status and symptoms of autism in children and adults. Nutritional and metabolic biomarkers were measured at the beginning and end of the study for a subset of the participants (53 children ages 5-16 years). The nutritional and metabolic status of those children at the start of the study (pre-supplementation) was compared with that of neurotypical children of similar age and gender and reported previously, as discussed above [20]. Three measures of autism severity were measured pre and post, and a fourth measure of change in autism symptoms was measured at the end of the study.

## Methods

The basic design of the study was a randomized, double-blind, placebo-controlled study lasting three months. This study was conducted with the approval of the Human Subjects Institutional Review Board of Arizona State University.

### Participants

The participants were recruited in two groups, an Arizona group and a National group. Both groups were treated identically, except that the Arizona group also participated in an extensive pre and post analysis of their nutritional and metabolic status, whereas the National group did not participate in any medical assessment. The Arizona group had a more narrow age range since some biomedical markers vary with age; the National group included children and adults, to determine if the effect of the supplement on symptoms depended on age. Participants were recruited from Arizona with the help of the Autism Society of America - Greater Phoenix Chapter and the Arizona Division of Developmental Disabilities. National participants were recruited with the help of the Autism Research Institute and the Autism Society of America. All parents and (where developmentally appropriate) children signed parent consent/child assent forms, and all adult participants signed for themselves (where developmentally appropriate) and/or their parents/guardians signed for them.

Unsupplemented neurotypical children, recruited as part of the preliminary baseline data collection [20] provided a reference range for nutritional and metabolic status for all of the measurements.

#### Enrollment criteria

1) Arizona: age 5-16 years; National: age 3-60 years old;

2) Prior diagnosis of autism, PDD/NOS, or Asperger's by a psychiatrist or similar professional, with written verification (no additional assessment was done in this study)

3) No usage of a vitamin/mineral supplement in the last 2 months

4) No changes in any medical, dietary, behavior, or other treatment in the last two months, and a willingness to avoid any changes during the study

5) No current use of any chelation treatment

#### Participants

Approximately 300 applications were received, and 41 applications were rejected, primarily due to current usage of vitamin/mineral supplements or (for the Arizona group) due to being outside the age range. For the Arizona group, 74 applications were approved, and 53 participants enrolled in the study. For the National group, 181 applications were approved, and 88 participants enrolled in the study. The primary reasons why some families chose not to enroll appeared to be primarily the chance of receiving the placebo, the amount of questionnaires to fill out, and (for the Arizona group) the blood and urine collections.

The characteristics of the study participants are listed in Table 1, and their symptoms and co-morbid symptoms are described in Table 2. In the national study, 41% of participants were from the West, 24% came from the South, 17% from the Midwest, 17% from Northeast and 1% from the Pacific.

**Table 1:** Characteristics of Participants

	Arizona		National Group	
	**Placebo**	**Supplement**	**Placebo**	**Supplements**

**Total Participants**	27	26	42	46

**Male**	22 (81%)	25 (96%)	39 (93%)	39 (85%)

**Female**	5 (19%)	1 (4%)	3 (7%)	7 (15%)

**Age (years)**	10.5 ± 3.1	9.3 ± 3.2	9.5 +/- 5.7	13.1 +/- 10.0

**Diagnosis**	74% Autism; 19% Aspergers; 7% PDD/NOS	96% Autism; 4% Aspergers	67% Autism 12% Asperger's 21% PDD/NOS	85% Autism 7% Asperger's 9% PDD/NOS

**Medications**	52% no medication; 8 (30%) psycho-pharmaceuticals - primarily risperdal and clonidine; 4 (15)% CNS stimulants (primarily Concerta); 1 (4%) anti-convulsants; 1 (4%) asthma/allergy medications; 1 (4%) GI medications; 1 (4%) Insulin medications; 2 (7%) blood pressure medications	42% no medication; 8 (32%) psycho-pharmaceuticals - primarily risperdal and clonidine; 1 (4%) CNS stimulants (Dextrostat); 1 (4%) anti-convulsants; 3 (12%) asthma/allergy medications; 2 (8%) GI meds	64% no medications; 10 (24%) atypical anti-psychotics (primarily Risperdal); 2 (5%) on CNS stimulants; 3 (7%) on anti-convulsants; 4 (10%) on allergy/asthma medications; 1 (2%) on GI medications	52% no medications; 14 (30%) atypical anti-psychotics (primarily risperdal); 3 (7%) on CNS stimulants; 7 (15%) on anti-convulsants (primarily Depakote); 6 (13%) on allergy/asthma medications; 1 (2%) on muscle relaxants; 1 (2%) on anti-coagulants

**Special Diets**	67% on regular diet; 3 gluten-free, casein-free diet; 2 reduced dairy; 2 low sugar	88% on regular diet; 1 gluten-free; 1 reduced gluten; 1 reduced dairy	76% on regular diet; 11% gluten-free, casein-free diet; 1 reduced gluten and casein diet; 2 milk-free; 1 soy-free 1 Feingold diet	67% on regular diet; 11% gluten-free, casein-free diet; 15% reduced dairy/gluten/casein; 1 dairy-free; 1 casein-free; 1 gluten-free; 1 low sugar; 1 no red/blue dyes; 1 lactose free; 1 low soy; 1 vegetarian; 1 no eggs

**Nutritional Supplements**	None	1 on fish oil; 3 on melatonin	5% on fish oil; 1 on melatonin; 1 digestive enzymes; 1 on glucosamine & chondroitin sulfate; 1 on constipation relief	4% on fish oil; 1 on glutamine; 1 on herbal sleep extract; 1 on multi-nutrient supplement

**Table 2:** Symptoms of Autism Participants, per the ATEC Subscale on Health/Physical Behavior.

Symptom	% with moderate or severe problem
bedwetting	20%

wets pants/diapers	18%

soils pants/diapers	21%

diarrhea	14%

constipation	24%

sleep problems	36%

eats too much/little	48%

limited diet	47%

hyperactive	40%

lethargic	9%

hits/injures self	20%

hits/injures others	18%

destructive	20%

sound sensitive	43%

anxious/fearful	30%

unhappy/crying	12%

seizures	2%

obsessive speech	27%

rigid routines	36%

shouts/screams	38%

demands sameness	34%

often agitated	31%

not sensitive to pain	30%

hooked or fixated on certain objects	62%

repetitive movements	43%

This section was rated on a scale of 0 (none), 1 (mild), 2 (moderate), 3 (severe). Below are listed the percentages with moderate or severe problems, as reported by parents.

#### Study Protocol

1) Participant parents contacted the study coordinator, and the study was explained by telephone. Consent/assent forms were sent to the parents for review, and then signed copies were mailed or brought to the study coordinator. Initial assessments of autism severity were conducted.

2) (Arizona only) The study physician conducted a physical exam to determine that the children were in adequate health for participating in the study.

3) (Arizona only) Morning blood samples were collected after an overnight fast (8-12 hours). Morning urine samples were collected, and in almost all cases these were first-morning (overnight) urines. Samples were sent in a blinded manner to the labs for testing.

4) Assignment and blinding: The study coordinator assigned subject code and randomized for group assignment prior to baseline data gathering. All other study personnel (nurses, physician, laboratory staff, and PI) remained blind to group assignment; study instructions for all subjects were identical and provided blind to group assignment.

5) Dosing and titration: Participants in both groups were given a liquid (supplement or placebo) to be administered in three equally divided doses with food at breakfast, lunch, and dinner. Dosing was calculated and administered on a volume basis (per ml) using supplied oral syringes. The dosage for all subjects was slowly titrated up to their full dose (see Dosage section below) over the first 3 weeks of the study (or longer if necessary).

6) Monitoring: Following collection of baseline data, participants were monitored throughout the study by telephone and/or email for individualized dosing titration and for potential adverse effects. This was done by the study nurse with supervision from the study physician both of whom were blind to group assignment. Monitoring decisions were made based on the assumption of subject being on verum. During initial dosing individualization, monitoring was done weekly, and then bi-weekly (or more often if needed) during the remainder of the study.

7) At the end of the study (3 months), final assessments of autism severity were conducted.

8) (Arizona only) At the end of the study, morning blood and urine samples were collected again.

For the neurotypical children, only steps 1-3 were followed - they did not participate in the treatment portion of the study.

### Supplement/Placebo Formulation

Both formulations were produced by Yasoo Health and passed USP < 51 >, the antimicrobial effectiveness test, and the supplement was analytically tested and found to meet label claims. Both were predominantly water-based, flavored with a natural cherry flavor and sweetened with sucralose, and both contained preservatives (potassium sorbate and sodium benzoate). The supplement also contained sucrose due to the strong flavor of the vitamins/minerals.

The placebo was 97% water and also contained a small amount of beta-carotene for coloring, and citric acid and a proprietary blend of natural plant-based flavors to create a vitamin-like after-taste. The citric acid and natural plant-based flavors were not included in the supplement. A small amount of xanthum gum was used to thicken the placebo, to simulate the viscosity of the supplement.

The placebo was packaged identically to the supplement, and based on the participants' discussions with the nurses (who were also blinded), it did not appear that the participants could distinguish if they had received the placebo or the supplement, based on taste.

### Supplement

The vitamin/mineral supplement formulation is given in Table 3, for a child of 60 lb; the dosage was adjusted up or down proportionately according to bodyweight (measured at the start of the study), up to a maximum of 100 pounds. As discussed earlier, it is a "second-generation" formulation, based on the results of a small unpublished pilot study. It is a comprehensive vitamin/mineral supplement, containing most vitamins and minerals. A comparison with the RDA/AI and Tolerable Upper Limit [21] is shown in Table 3. Two essential minerals, iron and copper, were not included because our preliminary data suggested they were not needed by most children with autism. The form of vitamin B6 used was pyridoxine, because that form can enter the cell and be converted into the active form, pyridoxal-5-phosphate (P5P); in contrast, P5P cannot enter cells [22]. The amount of vitamin B6 is moderately high compared to the RDA because in children with autism many B6-dependent biomarkers were known by prior research to be abnormally low, including glutathione and neurotransmitters. Methylsulfonylmethane (MSM, (CH_3_)_2_SO_2_) was included as a source of sulfate, because our pilot study found children with autism had very low levels of plasma sulfate. Lithium, a possibly essential mineral [23] was included because an earlier study [24] found that children with autism and their mothers were low in lithium, and low lithium is linked to a wide range of psychological disorders. Note that the dosage of lithium is similar to the typical daily intake, and less than 1% of the level when lithium is used as a psychiatric medication. Coenzyme Q-10 was added to support mitochondrial function. A low dose of N-acetyl-cysteine was included to enhance production of glutathione. This formulation contained a water soluble form of vitamin E (d-Alpha-Tocopheryl Polyethylene Glycol-1000 Succinate) that has shown to improve the absorption of fat-soluble vitamins in patients with malabsorption [25-28].

**Table 3:** Formulation of vitamin/mineral supplement used in present study, and comparison to Recommended Daily Allowance (RDA) or Adequate Intake (AI) and Tolerable Upper Limit.

VITAMINS	Current Supplement (for 60 lb child)	RDA/AI (4-8 yr) (AI values indicated by asterisk)	Upper Limit for children ages 4-8 years
**Vitamin A** (palmitate)	1000 IU	400 mcg (1333 IU)	900 mcg (3000 IU)

**Vitamin C** (calcium ascorbate)	600 mg	25 mg	650 mg

**Vitamin D3** (cholecalciferol)	300 IU	5 mcg (200 IU)*	50 mcg (2000 IU)

**Vitamin E**	150 IU	7 mg (10.5 IU)	300 mg (450 IU)

**Mixed Tocopherols**	70 mg	n/a	n/a

**Vitamin K**	0	55 mcg*	ND

**B1** (thiamin HCl)	20 mg	0.6 mg	ND

**B2** (riboflavin)	20 mg	0.6 mg	ND

**B3** **(niacin/niacinamide)**	15 mg niacin 10 mg niacinamide	8 mg	15 mg

**B5 **(calcium d-pantothenate)	15 mg	3 mg*	ND

**B6 **(pyridoxine HCl)	40 mg	0.6	40 mg

**B12 **(cyanocobalamin)	500 mcg	1.2 mcg	ND

**Folic Acid**	100 mcg	200 mcg	400 mcg

**Folinic Acid**	550 mcg		

**Biotin** (biotin)	150 mcg	12 mcg*	ND

**Choline** (choline chloride)	250 mg	250 mg*	1000 mg

**Inositol**	100 mg	n/a	n/a

**Mixed Carotenoids**	3.6 mg	n/a	n/a

**Coenzyme Q10**	50 mg	n/a	n/a

**N-acetyl cysteine**	50 mg	n/a	n/a

**MINERALS**			

**Calcium** (from calcium ascorbate)	100 mg	800 mg*	2500 mg

**Chromium** (chromium amino acid chelate)	70 mcg	15 mcg*	ND

**Copper**	0	440 mcg	3000 mcg

**Iodine** (potassium iodide)	100 mcg	90 mcg	300 mcg

**Iron**	0	10 mg	40 mg

**Lithium** (lithium orotate)	500 mcg	n/a***	n/a

**Magnesium** (magnesium chloride hexahydrate)	100 mg	130 mg	110 mg**

**Manganese** (manganese amino acid chelate)	3 mg	1.5 mg*	3 mg

**Molybdenum** (sodium molybdate dihydrate)	150 mcg	22 mcg	600 mcg

**Phosphorus**	0	500 mg	3000 mg

**Potassium** (potassium chloride)	50 mg	3.8 g*	n/a

**Selenium** (selenomethionine and sodium selenite)	22 mcg	30 mcg	150 mcg

**Sulfur** (MSM)	500 mg	n/a	n/a

**Zinc** (zinc gluconate)	12 mg	5 mg	12 mg

Other Ingredients in Current Supplement	Natural cherry flavor; sucrose, sucralose; preservatives (potassium sorbate, sodium benzoate).		

ND: none determined

* Adequate Intake

** for Magnesium, the UL is the amount for supplements and does not count food sources

*** Estimated daily intake of lithium in food is 1900 mcg/day for adults.

### Dosage

All participants (children and adults) received either the supplement or placebo, and the dosage was adjusted based on baseline measured body weight up to a maximum of 100 pounds (see Table 3). Based on prior studies dosage levels of nutrients in the supplement were chosen to be significantly higher than RDA levels, but either at or below the Tolerable Upper Limit. The supplement/placebo was administered by the parents (or school staff at lunchtime). Compliance was monitored by a daily checklist, and was above 95% in all cases.

The study dose was gradually increased during the first 3 weeks of the study:

Days 1-4: 1/6 of full dose

Days 5-8: 2/6 of full dose

Days 9-12: 3/6 of full dose

Days 13-16: 4/6 of full dose

Days 17-20: 5/6 of full dose

Days 21 and later: full dose.

The dosage was individually titrated in cases where parents reported possible adverse effects (see section on Withdrawals/Removals/Adverse Effects), with the dosage being lowered temporarily in some cases. By the end of the study, most participants were at the full dose, except for 2 children on the placebo and 6 children on the supplement (they ended at 50%-83% of the full dose). Thus, for most children the full dosage was well-tolerated, but for approximately 10% a slightly lower dosage was used to reduce or eliminate side-effects.

#### Lab Measurements

Blood and urine samples were sent in a blinded fashion to two laboratories, Vitamin Diagnostics and Doctor's Data, for evaluation. Details of the measurement methods are given in another paper [20]. Both laboratories are certified by CLIA, the Clinical Laboratory Improvement Amendments program operated by the US Department of Health and Human Services which oversees approximately 200,000 laboratories in the US, and the tests reported in the paper are CLIA-approved tests.

#### Assessing Autistic Symptoms and Severity

Three tools were used by the same parent/guardian at the beginning and end of the study to assess the severity and symptoms of autism, namely the Pervasive Development Disorder Behavior Inventory (PDD-BI) [29], Autism Evaluation Treatment Checklist (ATEC) [30], and Severity of Autism Scale (SAS) [31]. For the PDD-BI, we used a slightly modified Autism Composite, in which the Semantic/Pragmatic Problems (SemPP) subscale is ignored. The reason is that the SemPP is difficult to interpret, since children with no spoken language inappropriately score as less severe than those with limited language. Therefore, following the example of our previous study [31] we exclude the SemPP subscale in calculating the Autism Composite score, resulting in a modified Autism Composite score consisting of Sensory/Perceptual Approach, Ritualisms/Resistance to Change, Social Pragmatic Problems, Social Approach Behaviors, Phonological and Semantic Pragmatic subscales.

In addition, we used a revised form of the Parent Global Impressions (PGI-R), which we introduce here. It was evaluated at the end of the study only, since it only assesses changes in symptoms. The original Parent Global Impression (PGI) [14] was a simple list of 8 symptoms, including Expressive Language, Receptive Language, General Behavior, Gastrointestinal Symptoms, Sleep, Sociability, Eye Contact, and Overall. The symptoms were rated on a scale of 1-7, where 1 = much worse, 2 = worse, 3 = slightly worse, 4 = no change, 5 = slightly better, 6 = better, 7 = much better. The PGI-Revised replaces the "General Behavior" category in the PGI with the more specific categories of Hyperactivity, Tantrumming, Cognition, and Play. Also, an Average Change score is computed, based on the average of the individual scores. Finally, the scale is changed from a range of 1-7 to a range of -3 to +3; ie, -3 = much worse, 0 = no change, and +3 = much better.

#### Statistical Analysis

Several types of statistical analyses were used, depending on the research question being addressed. In comparing levels between groups (such as children with autism vs. neurotypical children), 2-sided unpaired t-tests were used. The unpaired t-tests were either done assuming equal variance (if p-values for F-tests for equal variance were greater than 0.05), or assuming unequal variance (if F-test p-value results were less than 0.05). For individual comparisons a p value of 0.05 or lower was assumed significant. However, when multiple comparisons were considered, then a lower p-value was considered significant based on a Bonferroni analysis - this is defined at the beginning of each section of the results. In other words, if one asks the question "did the level of vitamin B6 improve", a p-value of 0.05 is sufficient for 95% confidence. However, if one asks the question "did the level of any of the vitamins improve", then a Bonferroni correction is used. This study is exploratory in that we are investigating many hypotheses; i.e., will vitamin/mineral supplementation affect the level of vitamins, minerals, and metabolic factors. This is necessary because the supplement contains many vitamins and minerals, so it is expected to affect the levels of many of those, as well as other nutritional and metabolic markers that depend on them.

Pearson Correlation coefficients were obtained to determine the strengths of linear relationships among the variables involved in the analyses.

Note that for a few measurements there was some data below our detection limit. In those cases we substituted the value of the detection limit for the data point; so, for cases where some samples were below detection limit, our reported averaged values are an upper bound to the true average value.

In this paper we focus on the percentage change (pre to post) for each biomarker for the supplement group and for the placebo group separately. In most cases there were few significant changes in the placebo group, so supplement vs. placebo group comparisons were not made. Randomization of the ASD children sometimes resulted in somewhat different baseline values for some analytes. Thus in data analysis a paired t-test comparing child to self was chosen instead of unpaired t-test comparing the two groups.

Regression analysis was employed to examine the relationship between the Average Change of the PGI-R and the biomarkers of nutritional and metabolic status, for the Arizona supplement group only. For the selected dependent and independent variables, step-wise linear regression analyses were conducted. The initial variables were the variables with the strongest correlation to the PGI-R. Then at each step, the variable with the highest p-value was eliminated, and this process was continued until the adjusted R^2 ^value began declining. Thus, the goal was to determine the best fit to the sample data for the selected model, taking into account the correlation among the independent variables.

#### Participant Withdrawals, Removals, and Adverse Effects

**Figure 1:**
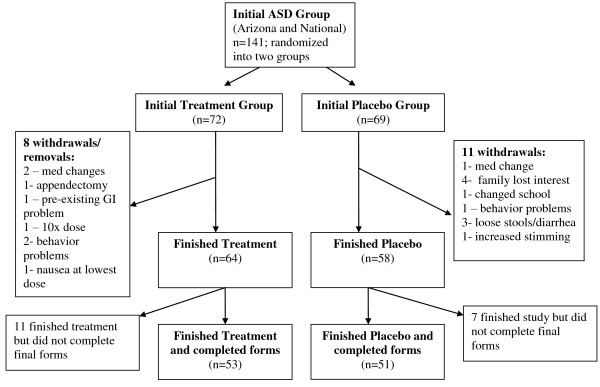
**Study Flow Chart**.

Figure 1 displays a flow chart of the study. Two of the children with autism from the initial baseline evaluation [20] did not start the supplement study. The withdrawals/removals included:

Placebo Group (11 withdrawals, including 5 due to adverse effects)

4 participants withdrew because their parents lost interest in the study

1 child was removed by the PI due to a change of school

1 participant began behavior medication 1 week into study and was removed by the PI

3 cases of loose stools/ diarrhea (all 3 had gut issues prior to starting, and the continuation of those symptoms caused them to drop out)

1 participant had increased stimming (rubbing face) - history of this, but seemed to worsen

1 participant withdrew due to behavior problems

Treatment Group (8 withdrawals, including 3 due to adverse effects)

2 participants were removed from the study because they made changes in their psychiatric medications in the first few weeks of the study.

1 participant dropped due to an appendectomy during the first week of the study.

1 participant was removed at the beginning of the study because the study physician judged that their initial gut problems (prior to starting the supplement) required immediate treatment which required exclusion from the study

1 participant was removed because their parent misunderstood the dosage and gave 10× the specified dosage for the first two weeks. The child was receiving the real supplement, and was doing very well with no adverse effects. They completed a Parent Global Impressions-Revised form and reported some of the highest improvements of any child in the study.

1 case of aggressive behavior, night terrors, trouble focusing - history of this, but seemed to worsen

1 case of aggressive behavior, moody - history of this, but seemed to worsen

1 case of nausea/diarrhea at lowest dose (person had long history of having similar reactions to almost all vitamin supplements)

Some mild temporary adverse effects were reported in both the supplement and placebo group, generally related to mild behavior problems (approximately 11% and 7%, respectively) or diarrhea/constipation (approximately 11% and 7%, respectively), but did not cause participants to withdraw. Most adverse effects were encountered during individualized titration and resolved by slowing the rate of increase to full dose or lowering the dosage (see dosage section) of the supplement/placebo. These reports occurred in both the supplement and placebo group, so some were probably due to normal fluctuation in existing autistic symptoms.

In the National group only, there were 18 participants who completed the study but did not fill out the final evaluation forms despite several requests (7 cases in the placebo group, and 11 cases in the supplement group). This did not occur in the Arizona group, because those families filled out forms when they returned for their final blood draws.

Combining the Arizona and National groups, 141 children and adults started the study, 19 withdrew, 18 did not complete final paperwork, and 104 completed the study and filled out the final evaluations, with 51 in the placebo group and 53 in the supplement group.

## Results

### Nuritional/Metabolic Results

In this section we discuss the results for the Arizona participants who began and ended the study, including 21 in the Treatment group and 24 in the Placebo group. However, in a few cases blood or urine measurements were not available both pre and post, due to problems including compliance with blood and urine collection, incomplete blood draws, and loss of samples due to shipping or laboratory errors. The tables specify the number of complete cases for each category of measurements; incomplete cases (lack of data for beginning or end of study) are not included in those tables or in the analysis. In all tables the values for the Neurotypical Controls (N = 44) from the preliminary phase of the overall study are included as a contemporaneous reference range; the samples from the neurotypical controls were obtained in the same sessions as for the autism group, in an identical manner, and shipped together in a blinded fashion to the laboratories for testing.

### Vitamins

**Figure 2:**
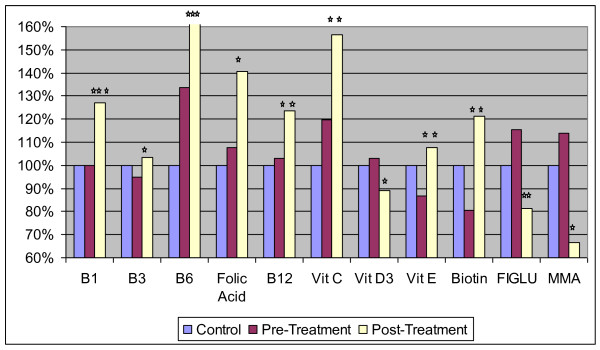
**Level of vitamins and related substances in neurotypical controls and in the Arizona autism treatment group (pre and post), normalized to the level in the control group**. The average values are shown. The number of asterisks indicates the p-value for the t-test of the change in the biomarker from pre-treatment to post-treatment in the autism group (* p < 0.05, ** p < 0.01, *** p < 0.001). Note that the post-treatment value for Vitamin B6 is off the scale.

Table 4 shows the levels of vitamins and related substances, and Figure 2 shows the significant changes for the treatment group. There are a total of 21 comparisons, so in this section statistically "significant" is defined as p < 0.002, "marginally significant" as p < 0.005, and "possibly significant" as p < 0.05.

**Table 4:** Vitamins: The average levels of vitamins measured in the Neurotypical group and the Autism Treatment and Autism Placebo groups (pre and post) who completed the study are reported below, along with their standard deviations.

Vitamins	Units	Neuro-typicals (n = 44)	Arizona Treatment Group (N = 18)				Arizona Placebo Group (N = 22)			
			**Pre**	**Post**	**% change**	**p-value**	**Pre**	**Post**	**% change**	**p-value**

Vit. A (plasma)	ug/100 ml	54.9 +/- 12	62.3 +/- 12	59.0 +/-13	-5%	n.s.	50.2 +/- 7.2	52.9 +/-13	+5%	n.s.

Total Carotenes (beta carotene and other carotenes, in plasma)	ug/ 100 ml	178 +/-53	158 +/- 53	170 +/- 65	+7%	n.s.	136 +/- 53	150 +/-59	+10%	n.s.

Vit B1 Thiamine (WB)	ug/l	63 +/-9	**63** **+/- 12**	**80** **+/-12**	**+27%**	**0.0005**	65 +/-9	65 +/-11	0%	n.s.

Vit B2 Riboflavin (WB)	ug/l	282 +/-52	291 +/- 42	295 +/-36	1%	n.s.	272 +/- 45	262 +/-55	-3%	n.s.

Vit B3 Niacin and Niacinamide (WB)	ug/l	7.07 +/-1.0	**6.7** **+/- 1.1**	**7.3** **+/-0.9**	**+9%**	**0.04**	7.1 +/- 1.3	7.2 +/-1.0	+1%	n.s.

Vit B5 Pantothenic Acid (WB)	ug/l	714 +/-180	654 +/- 104	748 +/-170	+14%	0.06	600 +/- 117	629 +/-159	+5%	n.s.

Vit B6 (as P5P in RBC)	ug/l	15.2 +/-5.3	20.3 +/- 15	**58.1** **+/-24**	**+187%**	**0.00001**	17.8 +/- 14	16.1 +/-11	-10%	n.s.

Folic Acid (serum)	ug/l	18.7 +/-6.1	20.1 +/- 7.5	**26.3** **+/-7.1**	**+31%**	**0.03**	15.3 +/- 4.8	17.8 +/-8.2	17%	0.09

Vit B12 (plasma)	ng/l	676 +/-215	**696** **+/- 231**	**835** **+/-259**	**+20%**	**0.002**	690 +/- 240	639 +/-246	-7%	n.s.

Vit C (plasma)	mg/ 100 ml	1.33 +/-0.46	**1.59** **+/- 0.60**	**2.08** **+/-0.34**	**+31%**	**0.007**	1.60 +/- 0.59	1.55 +/-0.51	-3%	n.s.

Vit D**3** (25-hydroxy in plasma)	ug/l	28.6+/-8.5	29.6 +/- 8.3	**25.4** **+/-6.2**	**-14%**	**0.04**	28.9 +/- 8.7	25.9 +/-7.8	-10%	n.s.

Total Vit E (serum)	mg/ 100 ml	0.90 +/-0.32	**0.78** **+/- 0.16**	**0.97** **+/-0.21**	**+24%**	**0.002**	0.75 +/- 0.19	0.84 +/-0.24	+12%	n.s.

Biotin (WB)	ng/l	491 +/-164	**395** **+/- 115**	**595** **+/-225**	**+51%**	**0.008**	**377** **+/- 88**	**527** **+/-162**	**+40%**	**0.001**

Vit K (plasma)	nmol/l	295 +/- 189	288 +/- 116	330 +/- 165	+15%	n.s.	317 +/- 197	277 +/- 98	-13%	n.s.

**Vitamin-like substances**										

Free Choline (RBC)	mg/l	5.6 +/- 1.7	6.7 +/- 3.5	5.9 +/- 2.0	-12%	n.s.	6.4 +/- 2.3	7.0 +/- 1.7	+10%	n.s.

Total Choline (RBC)	mg/l	310 +/- 51.4	363 +/- 59	343 +/- 43	-5%	n.s.	361 +/- 67	368 +/- 42	+2%	n.s.

Lipoic Acid (plasma)	ug/l	2.9 +/- 1.2	3.1 +/- 1.8	2.3 +/- 0.7	-22%	n.s.	2.3 +/- 1.5	2.1 +/- 1.2	-9%	n.s.

**Biomarkers of functional need for vitamins**										

FIGLU	ug/l	1.62 +/- 0.72	**1.87** **+/- 0.93**	**1.32** **+/- 0.64**	**-29%**	**0.01**	2.11 +/- 0.91	1.85 +/- 0.98	-12%	n.s.

Methylmalonic Acid	mg/g-creat	7.2 +/- 4.8	**8.2** **+/- 5.6**	**4.8** **+/- 4.2**	**-41%**	**0.03**	6.7 +/- 5.7	8.7 +/- 10.4	+30%	n.s.

N-methyl-nicotinamide	mg/g-creat	3.44 +/- 2.1	4.62 +/- 3.5	3.1 +/- 1.6	-33%	n.s.	4.94 +/- 5.1	5.15 +/- 3.1	+4%	n.s.

Kryptopyrroles	ug/dl	35.8 +/- 15	38.0 +/- 23	36.2 +/- 29	-5%	n.s.	40.3 +/- 29	38.1 +/- 24	-5%	n.s.

The p-value for a t-test comparison of the change in the level is reported. If the p-value is below 0.05 then the result is highlighted.

#### Treatment Group

After supplementation for 3 months, the supplement group had significant increases (p < 0.002) in vitamins B1, B6, B12, and E. There were possibly significant (p < 0.05) increases in vitamin B3, C, biotin, and folic acid, and a possible decrease in vitamin D (due to seasonal effects -see discussion section). There were also possibly significant decreases in FIGLU and methylmalonic acid, indicating that the need for folic acid and vitamin B12, respectively, had been met.

#### Placebo Group

There was a very significant increase in biotin (p < 0.001), but no other significant or possibly significant changes in vitamins. The increase in biotin may be due to fluctuations in diet, seasonal changes, or the plant-based extract which was used in the placebo (not the supplement) to give it a "vitamin-like" flavor.

### Essential Minerals

Table 5 shows the levels of minerals in whole blood (WB), RBC, serum, and urine (for iodine) for the study participants. There are a total of 30 comparisons, so in this section statistically "significant" is defined as p < 0.0017, "marginally significant" as p < 0.0034, and "possibly significant" as p < 0.05.

**Table 5:** Minerals: The average levels of minerals measured in the Neurotypical group and the Arizona Autism Treatment and Arizona Autism Placebo groups (pre and post) who completed the study are reported below, along with their standard deviations.

Essential Minerals + other minerals	Neuro-typicals (n = 44)	Treatment Group (n = 19)				Placebo Group (n = 20)			
		**Pre**	**Post**	**% change**	**p-value**	**Pre**	**Post**	**% change**	**p-value**

Calcium-WB (mg/dL)	5.8 +/- 0.3	5.9 +/- 0.4	6.0 +/0.4	+1%	n.s.	5.8 +/- 0.3	5.6 +/- 0.5	-2%	n.s.

Calcium-RBC (ug/g)	22.4 +/- 6	**19.2** **+/- 7.9**	**27.4** **+/- 3.6**	**+43%**	**0.001**	**19.5** **+/- 7.5**	**25.2** **+/- 4.3**	**+30%**	**0.02**

Calcium-Serum (mg/dL)	9.60 +/- 0.2	9.65 +/- 0.4	9.51 +/- 0.3	-1%	0.07	9.59 +/- 0.4	9.53 +/- 0.3	0%	n.s.

Chromium-RBC (ng/g)	0.80 +/- 0.4	1.02 +/- 0.50	0.81 +/- 0.6	-21%	n.s.	0.77 +/- 0.44	0.70 +/- 0.3	-9%	n.s.

Copper-WB (ug/dL)	89 +/- 14	91.2 +/- 13	95.9 +/- 17	+5%	0.09	93.9 +/- 10	95.5 +/- 13	+2%	n.s.

Copper-RBC (ug/g)	0.72 +/- 0.09	0.75 +/- 0.08	0.78 +/- 0.08	+4%	0.06	0.74 +/- 0.08	0.77 +/- 0.08	+4%	n.s.

Iodine-Urine (ug/mg creatinine)	0.26 +/- 0.2	**0.28** **+/- 0.26**	**0.43** **+/- 0.27**	**+54%**	0.03*	0.23 +/- 0.15	0.22 +/- 0.15	-5%	n.s.

Iron-RBC (ug/g)	833 +/- 64	**887** **+/- 101**	**806** **+/- 54**	**-9%**	**0.006**	**883** **+/- 80**	**826** **+/- 51**	**-7%**	**0.02**

Iron-Serum (ug/dL)	87 +/- 35	91 +/- 34	88 +/- 46	-3%	n.s.	80 +/- 33	81 +/- 25	+2%	n.s.

Serum Ferritin	36.9 +/- 17	42.1 +/- 23	37.3 +/- 23	-11%	n.s.	**41.3** **+/- 25.1**	**33.4** **+/- 22**	**-19%**	**0.004**

Lithium-WB (ug/L)	3.6 +/- 6	**1.82** **+/- 0.8**	**10.6** **+/-5.1**	**+485%**	**.000001**	1.60 +/- 0.7	1.47 +/- 0.7	-8%	n.s.

Magnesium-WB (mg/dL)	3.64 +/- 0.26	**3.44** **+/- 0.19**	**3.62** **+/- 0.25**	**+5%**	**0.00007**	**3.65** **+/- 0.41**	**3.81** **+/-0.42**	**+4%**	**0.03**

Magnesium-RBC (ug/g)	47.5 +/- 5	**48.6** **+/- 7.5**	**44.9** **+/- 4.0**	**-8%**	**0.03**	49.4 +/- 5.8	47.9 +/- 6.0	-3%	n.s.

Magnesium-Serum (mg/dL)	2.03 +/- 0.15	1.90 +/- 0.13	1.95 +/- 0.15	+2%	n.s.	1.93 +/- 0.14	1.97 +/- 0.15	+2%	n.s.

Manganese-WB (ug/L)	11.6 +/- 3	**11.0** **+/- 3.3**	**13.6** **+/- 4.4**	**+23%**	**0.0006**	**12.0** **+/- 4.7**	**14.5** **+/- 5.0**	**+21%**	**0.00002**

Manganese-RBC (ug/g)	0.018 +/- 0.005	0.019 +/- 0.007	0.019 +/- 0.006	+1%	n.s.	0.020 +/- 0.006	0.019 +/- 0.007	-4%	n.s.

Molybdenum-WB (ug/L)	1.39 +/- 0.3	**1.27** **+/- 0.23**	**1.88** **+/- 0.9**	**+48%**	0.007*	1.34 +/- 0.3	1.37 +/- 0.3	+3%	n.s.

Molybdenum-RBC (ng/g)	0.98 +/- 0.2	1.01 +/- 0.3	1.32 +/- 0.0007	+31%	0.06*	0.87 +/- 0.26	0.93 +/- 0.2	+7%	n.s.

Phosphorus-RBC (ug/g)	567 +/- 43	**587** **+/- 73**	**546** **+/- 39**	**-7%**	**0.01**	**598** **+/- 48**	**564** **+/-37**	**-6%**	**0.002**

Phosphorus-Serum (mg/dL)	4.58 +/- 0.5	4.52 +/- 0.5	4.47 +/- 0.6	-1%	n.s.	4.57 +/- 0.5	4.68 +/- 0.7	+2%	n.s.

Potassium -RBC-mEq/L	76.9 +/- 4.1	78.1 +/- 6.2	75.5 +/- 4.4	-3%	n.s.	**81.1** **+/- 5.1**	**77.2** **+/- 5.1**	**-5%**	**0.01**

Potassium-Serum mEq/L	4.17 +/- 0.3	4.12 +/- 0.3	4.21 +/- 0.3	+2%	n.s.	4.01 +/- 0.3	4.04 +/- 0.3	1%	n.s.

Selenium-WB (ug/L)	210 +/- 20	**209** **+/- 25**	**223** **+/- 28**	**+7%**	**0.001**	**211** **+/- 39**	**218** **+/- 44**	**+3%**	**0.05**

Selenium-RBC (ug/g)	0.23 +/- 0.03	**0.251** **+/- 0.025**	**0.235** **+/- 0.03**	**-6%**	**0.007**	**0.244** **+/- 0.052**	**0.221** **+/- 0.04**	**-10%**	**0.002**

Sodium-Serum mEq/L	137 +/- 1	137 +/- 2	137 +/- 1	0%	n.s.	138 +/- 3	138 +/- 2	0%	n.s.

Vanadium-RBC (ng/g)	0. 22 +/- 0.07	0. 22 +/- 0.09	0. 21 +/- 0.05	-5%	n.s.	0.21 +/- 0.04	0.23 +/- 0.09	+10%	n.s.

Zinc-WB (ug/dL)	555 +/- 74	546 +/- 70	556 +/- 68	+2%	n.s.	559 +/- 59	567 +/-59	+1%	n.s.

Zinc-RBC (ug/g)	8.9 +/- 1.4	9.1 +/- 1.5	8.6 +/- 1.2	-5%	n.s.	9.1 +/- 1.1	8.8 +/- 1.2	-3%	n.s.

**Non-essential minerals**									

Boron-RBC (ug/g)	0.025 +/- 0.007	**0.031** **+/- 0.015**	**0.022** **+/- 0.008**	**-30%**	**0.004**	0.025 +/- 0.013	0.019 +/- 0.010	-24%	n.s.

Strontium-WB (ug/L)	24 +/- 6	21.6 +/- 3.7	22.8 +/-4.5	+6%	n.s.	29.1 +/- 10	28.6 +/-10	-2%	n.s.

Biomarker data was obtained only for the Arizona autism group. The p-value for a t-test comparison of the change in the level is reported. If the p-value is below 0.05, then the result is highlighted.

* The ttest for urinary iodine and WB & RBC Molybdenum was done after a square root transformation of the data, since the initial data was not close to a Gaussian distribution.

#### Treatment Group

**Figure 3:**
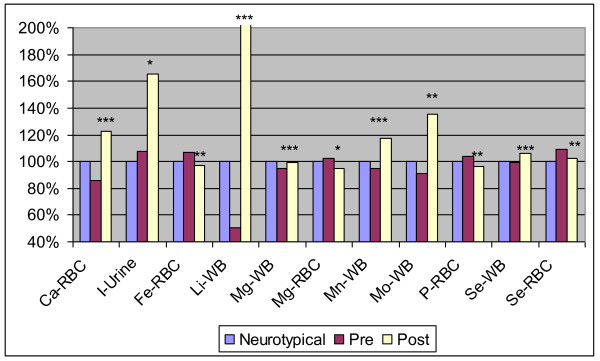
**Level of essential minerals in neurotypical group and in the Arizona autism treatment group (pre and post), normalized to the neurotypical group**. The average values are shown. The number of asterisks indicates the p-value for the t-test of the change in the biomarker from pre-treatment to post-treatment in the autism group (* p < 0.05, ** p < 0.01, *** p < 0.001). Note that the post-treatment value for lithium is off the chart. The figures uses standard abbreviations for the minerals, namely: Ca-calcium; I- iodine; Fe-iron; Li - lithium; Mg - magnesium; Mn - manganese; Mo - molybdenum; P-phosphorus; Se - Selenium.

Overall, there were many significant and possibly significant improvements in essential minerals - see Table 5 and Figure 3. The major improvements were significant large increases in WB lithium, WB manganese, and RBC calcium. There was a large and possibly significant increase in urinary iodine and WB molybdenum. There was a possibly significant decrease (improvement) in RBC iron, from a level above the average of the neurotypicals to a level slightly lower than the neurotypical average. There was a similar, but non-significant, decrease in serum ferritin. In all, supplementation tended to normalize the minerals, i.e. increasing if low and decreasing if high in comparison to control reference range.

There were also some minor changes. There was a small, statistically significant increase in WB magnesium but a small decrease in RBC magnesium that was possibly significant. Similarly there was a small, statistically significant increase in WB selenium, but a possibly significant decrease in RBC selenium (note that the concentration of selenium in WB is vastly greater than the concentration in RBC). There was a possibly significant small decrease in RBC phosphorus, from slightly high to slightly low, but no change in serum phosphorus. There was also a marginally significant decrease in boron, a non-essential mineral.

#### Placebo Group

Overall, it appears there was a significant increase in WB manganese, and mostly small fluctuations around average levels in the other minerals. There was a small marginally significant decrease in RBC selenium, and a possibly significant very small increase in WB selenium. There was a marginally significant small decrease in RBC phosphorus, from slightly high to slightly low. There were possibly significant increases in RBC calcium (from slightly low to slightly high) and WB magnesium (from average to slightly high), and possibly significant decreases in RBC potassium (from slightly high to average) and RBC iron (high to average).

### Sulfation, Methylation, Glutathione and Oxidative Stress

Table 6 shows the results for biomarkers of sulfation (free and total sulfate), methylation (SAM and uridine), glutathione (GSH), and oxidative stress (ratio of GSH:GSSG and nitrotyrosine). There are a total of 11 comparisons, so in this section statistically "significant" is defined as p < 0.005, "marginally significant" as p < 0.009, and "possibly significant" as p < 0.05.

**Table 6:** Sulfation, Methylation, Glutathione, and Oxidative Stress: The average levels measured in the Neurotypical group and the Autism Treatment and Autism Placebo groups (pre and post) who completed the study are reported below, along with their standard deviations.

		Neuro-typicals (n = 44)	Arizona Treatment Group (n = 18)				Arizona Placebo Group (n = 22)			
			**Pre**	**Post**	**% change**	**p-value**	**Pre**	**Post**	**% change**	**p-value**

Free Sulfate (plasma)	**umol/** **g-protein**	4.09 +/- 2.3	**1.60** **+/- 0.6**	**2.93** **+/- 2.0**	**+83%**	**0.008**	1.30 +/- 0.4	1.98 +/- 1.8	+52%	0.08

Total Sulfate (plasma)	**umol/** **g-protein**	1566 +/- 384	**1150** **+/- 254**	**1346** **+/- 236**	**+17%**	**0.001**	1093 +/- 184	1163 +/- 194	+6%	0.06

SAM (RBC)	umol/dl	228.4 +/- 12	**218** **+/- 17**	**230** **+/- 16**	**+6%**	**0.003**	**213** **+/- 12**	**218** **+/- 13**	**+3%**	**0.005**

SAH (RBC)	umol/dl	42.6 +/- 4.4	40.7 +/- 7.4	41.4 +/- 5.0	+2%	n.s.	**48.5** **+/- 6.9**	**45.4** **+/- 7.1**	**-6%**	**0.03**

Uridine (plasma)	10^-6 ^M	7.9 +/- 2.7	**16.2** **+/- 9.5**	**10.4** **+/- 4.7**	**-36%**	**0.008**	16.4 +/- 6.9	14.5 +/- 5.8	-11%	0.06

Adenosine (plasma)	10^-8 ^M	20.6 +/- 3.4	21.2 +/- 5.3	21.0 +/- 3.2	-1%	n.s.	**24.5** **+/- 6.2**	**22.8** **+/- 4.2**	**-7%**	**0.05**

Inosine (plasma)	10^-6 ^M	3.83 +/- 0.9	3.49 +/- 1.0	3.83 +/- 0.6	+10%	0.07	3.55 +/- 0.9	3.49 +/- 1.0	-2%	n.s.

Reduced plasma glutathione (GSH)	nmol/ml	4.09 +/- 0.79	**3.27** **+/- 0.59**	**3.84** **+/- 0.61**	**+17%**	**0.0008**	3.25 +/- 0.38	3.40 +/- 0.42	+5%	n.s.

Oxidized glutathione (GSSG)	nmol/ml	0.362 +/- 0.10	**0.467** **+/- 0.12**	**0.403** **+/- 0.09**	**-14%**	**0.02**	0.465 +/- 0.14	0.431 +/- 0.11	-7%	n.s.

Ratio of oxidized to reduced plasma glutathione		0.093 +/- 0.04	**0.150** **+/- 0.05**	**0.109** **+/- 0.03**	**-27%**	**0.002**	0.147 +/- 0.05	0.132 +/- 0.05	-10%	n.s.

Plasma nitro-tyrosine	ug/l	7.4 +/- 5.1	**14.1** **+/- 6.5**	**9.9** **+/- 5.4**	**-29%**	**0.004**	19.0 +/0 8.6	16.3 +/- 9.2	-14%	0.10

The p-value for a t-test comparison of the change in the level is reported. If the p-value is below 0.05, then the result is highlighted. Sulfation is assessed by free and total sulfate, methylation is assessed by SAM and uridine, glutathione is assessed by GSH, and oxidative stress is assessed by GSH:GSSG ratio and nitrotyrosine.

#### Treatment Group

After treatment, there was a significant increase in total sulfate, and a large and marginally significant increase in free sulfate. The level of SAM increased significantly, and there was a marginally significant decrease (improvement) in uridine, a marker of impaired methylation. Reduced glutathione improved significantly and nearly normalized. Two markers of oxidative stress, levels of nitrotyrosine and the ratio of oxidized:reduced glutathione significantly improved to near-normal levels. The level of oxidized glutathione improved to a near-normal level (possibly significant).

**Figure 4:**
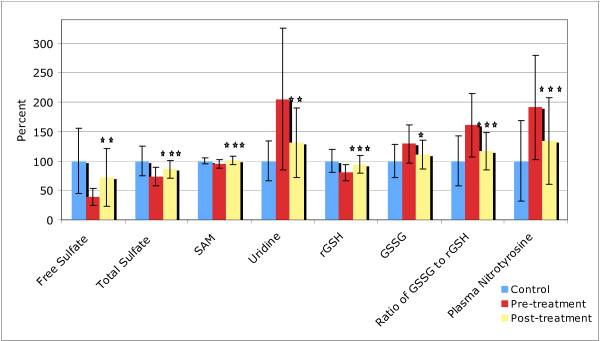
**Level of biomarkers in controls and in the Arizona autism treatment group (pre and post), normalized to the level in the neurotypical controls**. The average values and the standard deviations are shown. The number of asterisks indicates the p-value for the t-test of the change in the biomarker from pre-treatment to post-treatment in the autism group (* p < 0.05, ** p < 0.01, *** p < 0.001).

Figure 4 provides a comparison of the biomarkers that changed significantly from the beginning to the end of the study, normalized to the average level of the neurotypical group. In all cases there were improvements to normal or near-normal levels, which is one of the most significant findings of this study.

#### Placebo Group

There was a significant small increase in SAM, and a possibly significant small decrease in SAH and adenosine.

### ATP, NADH, NADPH, CoQ10

Table 7 shows the results for ATP, NADP, NADPH, and CoQ10. There are a total of 4 comparisons, so so in this section statistically "significant" is defined as p < 0.01, "marginally significant" as p < 0.025, and "possibly significant" as p < 0.05.

**Table 7:** ATP/NADH/NADPH/Co-Q10.

	Units	Neuro-typicals (n = 44)	Arizona Treatment Group (n = 18)				Arizona Placebo Group (n = 22)			
			**Pre**	**Post**	**% change**	**p-value**	**Pre**	**Post**	**% change**	**p-value**

ATP (plasma)	nmol/l	18.5 +/- 4.7	**15.5** **+/- 3.7**	**19.3** **+/- 2.3**	**+25%**	**0.00001**	14.0 +/- 4.9	15.6 +/- 3.7	+11%	0.08

NADH (RBC)	nmol/ml	20.7 +/- 4.3	**15.7** **+/- 4.5**	**20.1** **+/- 5.1**	**+28%**	**.00002**	15.3 +/- 3.9	16.4 +/- 4.1	+8%	0.06

NADPH (RBC)	nmol/ml	30.9 +/- 8.5	**23.0** **+/- 7.7**	**29.9** **+/- 7.1**	**+30%**	**0.001**	21.9 +/- 5.3	23.9 +/- 7.2	+9%	n.s.

CoQ10 (plasma)	ug/ml	0.60 +/- 0.16	**0.56** **+/- 0.14**	**1.41** **+/- 0.58**	**+153%**	**0.00001**	**0.57** **+/- 0.18**	**0.71** **+/- 0.26**	**+26%**	**0.01**

The average levels of biomarkers measured in the Neurotypical group and the Autism Treatment and Autism Placebo groups (pre and post) who completed the study are reported below, along with their standard deviations. The p-value for a t-test comparison of the change in the level is reported. If the p-value is below 0.05, then the result is highlighted.

#### Treatment Group

After supplementation, there was a large and very significant increase in the level of CoQ10, and the levels of ATP, NADH, and NADPH all increased very significantly to normal levels.

**Figure 5:**
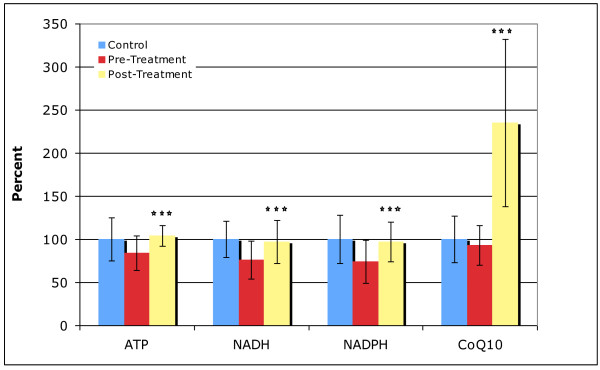
**Level of biomarkers in controls and in the Arizona autism treatment group (pre and post), normalized to the level in the neurotypical controls**. The average values and the standard deviations are shown. The number of asterisks indicates the p-value for the t-test of the change in the biomarker from pre-treatment to post-treatment in the autism group (* p < 0.05, ** p < 0.01, *** p < 0.001).

Figure 5 provides a comparison of the biomarkers that changed significantly from the beginning to the end of the study, normalized to the average level of the neurotypical group. ATP, NADH, and NADPH improved to normal levels, which is one of the most significant findings of this study.

#### Placebo Group

The level of CoQ10 increased slightly, and the increase was significant. The levels of ATP, NADH, and NADPH slightly improved, but the improvements were not significant.

### Primary Plasma Amino Acids

The levels of the primary (proteinogenic) plasma amino acids are given in Table 8. There are a total of 20 comparisons, so in this section statistically "significant" is defined as p < 0.0025, "marginally significant" as p < 0.005, and "possibly significant" as p < 0.05.

**Table 8:** Primary Amino Acids.

Amino Acids	Neuro-typicals (n = 44)	Arizona Treatment Group (n = 18)				Arizona Placebo Group (n = 20)			
		**Pre**	**Post**	**% change**	**p-value**	**Pre**	**Post**	**% change**	**p-value**

Histidine	8.2 +/- 1.3	8.7 +/- 2.5	9.6 +/- 1.3	+10%	n.s.	8.8 +/0 1.5	8.0 +/- 1.9	-9%	n.s.

Isoleucine	5.8 +/- 1.6	5.3 +/- 1.3	5.7 +/- 1.3	+8%	n.s.	**5.2** **+/- 0.8**	**6.0** **+/- 1.5**	**16%**	**0.03**

Leucine	10.7 +/- 2.1	11.0 +/- 2.5	11.4 +/- 2.8	+4%	n.s.	10.5 +/- 2.0	10.4 +/- 1.8	-1%	n.s.

Lysine	14.5 +/- 4.8	12.6 +/- 3.8	14.8 +/- 4.1	+17%	0.10	14.2 +/- 3.5	14.9 +/- 2.8	+5%	n.s.

Methionine	1.75 +/- 0.34	1.84 +/- 0.57	2.02 +/- 0.48	+10%	n.s.	1.92 +/- 0.45	1.93 +/- 0.64	+1%	n.s.

Phenylalanine	4.83 +/- 0.83	4.61 +/- 0.55	4.68 +/- 1.0	+2%	n.s.	4.36 +/- 0.63	4.65 +/- 0.66	+7%	n.s.

Threonine	8.88 +/- 2.1	9.2 +/- 2.6	7.9 +/- 1.8	-14%	0.07	9.4 +/- 3.6	8.7 +/- 2.4	-7%	n.s.

Tryptophan	4.33 +/- 1.0	3.32 +/- 0.9	4.11 +/- 1.7	+24%	0.06	**3.47** **+/- 1.1**	**4.17** **+/- 1.3**	**+20%**	**0.04**

Valine	20.5 +/- 4.3	20.6 +/- 4.3	20.0 +/- 4.2	-3%	n.s.	18.4 +/- 3.5	20.2 +/- 3.7	+10%	0.06

									

Alanine	33.4 +/- 8.9	36.6 +/- 9.0	37.4 +/- 12	+2%	n.s.	36.1 +/- 10	36.8 +/- 11	+2%	n.s.

Arginine	6.7 +/- 1.8	**5.9** **+/- 2.2**	**7.5** **+/- 1.6**	**+26%**	**0.006**	**6.9** **+/- 2.0**	**7.8** **+/- 1.5**	**+14%**	**0.02**

Asparagine	4.38 +/- 0.83	4.50 +/- 1.0	4.15 +/- 1.0	-8%	n.s.	4.26 +/- 1.42	3.92 +/- 0.84	-8%	n.s.

Aspartate	0.82 +/- 0.39	0.83 +/- 0.37	0.88 +/- 0.33	+6%	n.s.	0.69 +/- 0.21	0.82 +/- 0.37	+18%	n.s.

Cystine + cysteine	3.48 +/- 0.74	3.57 +/- 0.72	3.36 +/- 1.2	-6%	n.s.	3.01 +/- 0.81	3.40 +/- 0.87	+13%	n.s.

Glutamate	5.83 +/- 1.8	6.9 +/- 1.5	7.66 +/- 1.5	+12%	n.s.	6.4 +/- 1.5	6.6 +/- 1.5	+4%	n.s.

Glutamine	41.3 +/- 6.8	43.1 +/- 8.3	38.2 +/- 5.9	-11%	.08	42.4 +/- 11	38.4 +/- 5.0	-10%	0.10

Glycine	27.3 +/- 10	**23.8** **+/- 6.5**	**28.6** **+/- 6.7**	**+20%**	**0.02**	28.9 +/- 11	29.3 +/- 9.1	+1%	n.s.

Proline	15.8 +/- 4.9	15.9 +/- 4.2	16.0 +/- 4.9	+1%	n.s.	15.3 +/- 6.6	16.6 +/- 5.7	+8%	n.s.

Serine	9.5 +/- 2.1	**9.9** **+/- 2.1**	**8.9** **+/- 1.5**	**-10%**	**0.01**	**10.6** **+/- 2.1**	**9.7** **+/- 1.8**	**-8%**	**0.01**

Tyrosine	6.1 +/- 1.6	5.5 +/- 1.3	5.9 +/- 1.3	+6%	n.s.	5.6 +/- 1.1	6.0 +/- 1.3	+8%	n.s.

The average levels of primary amino acids in plasma (in units of micromoles per 100 ml) measured in the Neurotypical group and the Autism Treatment and Autism Placebo groups (pre and post) who completed the study are reported below, along with their standard deviations. The p-value for a t-test comparison of the change in the level is reported. If the p-value is below 0.05, then the result is highlighted.

#### Treatment Group

After supplementation, there were no significant or marginally significant changes. There were possibly significant increases in arginine and glycine, with both changing from slightly below the average neurotypical value to slightly above. There was a possibly significant decrease in serine, changing from slightly above the average neurotypical value to slightly below. In summary, all changes involved fluctuations about the normal value.

#### Placebo Group

There were no significant or marginally significant changes. There were possibly significant increases in arginine, isoleucine, and tryptophan, and a possibly significant decrease in serine.

### Secondary Plasma Amino Acids

The levels of secondary (non-proteinogenic) plasma amino acids are given in Table 9. Cystathione was also measured, but all the measurements except one were below the detectable limit of 0.05 umoles/100 ml, so those values are not listed. There are a total of 21 comparisons, so in this section statistically "significant" is defined as p < 0.0024, "marginally significant" as p < 0.0048, and "possibly significant" as p < 0.05.

**Table 9:** Secondary Amino Acids.

Amino Acids	Neuro-typicals (n = 44)	Arizona Treatment Group (n = 18)				Arizona Placebo Group (n = 20)			
		**Pre**	**Post**	**% change**	**p-value**	**Pre**	**Post**	**% change**	**p-value**

1-Methyl histidine	0.355 +/- 0.12	0.354 +/- 0.15	0.369 +/- 0.10	+4%	n.s.	**0.407** **+/- 0.14**	**0.329** **+/- 0.094**	**-19%**	**0.05**

3-Methyl histidine	0.68 +/- 0.52	0.68 +/- 0.55	0.54 +/- 0.46	-21%	n.s.	0.83 +/- 0.88	0.470 +/- 0.35	-43%	0.10

Alpha-amino adipate (31%/27% below dl)	0.088 +/- 0.044	0.084 +/- 0.035	0.099 +/- 0.052	+17%	n.s.	0.079 +/- 0.031	0.095 +/- 0.053	+20%	n.s.

Alpha-amino-N-butyrate	1.82 +/- 0.72	1.81 +/- 0.60	1.55 +/- 0.52	-15%	n.s.	1.68 +/- 0.58	1.60 +/- 0.50	-5%	n.s.

Anserine (84%/95% below dl)	0.051 +/- 0.005	0.055 +/- 0.019	0.0054 +/- 0.016	-1%	n.s.	0.052 +/- 0.0078	All results below detectable limit	-3%	n.s.

Beta-alanine	0.62 +/- 0.31	0.57 +/- 0.23	0.60 +/- 0.18	+6%	n.s.	0.72 +/- 0.35	0.58 +/- 0.16	-20%	n.s.

Beta-amino isobutyrate	0.138 +/- 0.088	0.170 +/- 0.070	0.162 +/- 0.077	-5%	n.s.	0.172 +/- 0.077	0.170 +/- 0.072	-1%	n.s.

Carnosine (73%/84% below dl)	0.054 +/- 0.015	0.063 +/- 0.042	(All results below dl)	-21%	n.s.	0.056 +/- 0.020	0.050 +/- 0.001	-11%	n.s.

Citrulline	3.02 +/- 0.52	3.02 +/- 0.69	3.34 +/- 0.98	+10%	n.s.	3.02 +/- 1.0	3.15 +/- 0.80	+4%	n.s.

Ethanol amine	0.94 +/- 0.63	1.15 +/- 0.90	0.81 +/- 0.47	-29%	n.s.	0.90 +/- 0.68	0.86 +/- 0.41	-4%	n.s.

Gamma-amino butyrate (75%/84% below dl)	0.053 +/- 0.008	0.053 +/- 0.08	0.053 +/- 0.007	-1%	n.s.	0.053 +/- 0.006	0.051 +/- 0.004	-3%	n.s.

Homocystine + Homocysteine (69%/86% below dl)	0.0055 +/- 0.0016	**0.0087** **+/- 0.0051**	**0.0057** **+/- 0.0014**	**-34%**	**0.02**	0.0078 +/- 0.0068	0.0054 +/- 0.0010	-30%	n.s.

Hydroxy proline	2.19 +/- 0.80	**2.34** **+/- 0.90**	**1.92** **+/- 0.58**	**-18%**	**0.03**	**2.54** **+/- 0.63**	**2.15** **+/- 0.51**	**-15%**	**0.04**

Methionine Sulfoxide	0.315 +/- 0.21	0.371 +/- 0.22	0.335 +/- 0.16	-10%	n.s.	0.341 +/- 0.15	0.432 +/- 0.30	+27%	n.s.

Ornithine	6.0 +/- 1.7	**5.0** **+/- 1.2**	**6.5** **+/- 1.9**	**+29%**	**0.008**	6.3 +/- 1.8	6.00 +/- 1.4	-5%	n.s.

Phospho ethanol amine	1.55 +/- 0.58	1.32 +/- 1.0	1.53 +/- 0.55	+16%	n.s.	1.51 +/- 0.56	1.80 +/- 0.56	+19%	n.s.

Phospho serine	0.024 +/- 0.044	0.055 +/- 1.0	0.015 +/- 0.007	-73%	n.s.	**0.058** **+/- 0.08**	**0.017** **+/- 0.009**	**-70%**	**0.05**

Sarcosine	0.89 +/- 0.36	**0.78** **+/- 0.32**	**1.06** **+/- 0.35**	**+35%**	**0.01**	**0.85** **+/- 0.34**	**1.12** **+/- 0.38**	**+32%**	**0.01**

Taurine	16.8 +/- 5.8	13.7 +/- 6.1	17.7 +/- 5.7	+29%	0.07	**15.5** **+/- 5.8**	**19.9** **+/- 5.9**	**+28%**	**0.03**

Urea	309 +/- 87	329 +/- 105	**270** **+/- 62**	**-18%**	**0.04**	257 +/- 71	252. +/- 106	-2%	n.s.

The average levels of secondary amino acids in plasma (in units of micromoles per 100 ml) measured in the Neurotypical group and the Autism Treatment and Autism Placebo groups (pre and post) who completed the study are reported below, along with their standard deviations. The p-value for a t-test comparison of the change in the level is reported. If the p-value is below 0.05, then the result is highlighted. In some cases data was below the detectable limit -if this was greater than 20%, then we report the data as the % of the autism group and then the % of the neurotypical group below the detectable limit (dl).

### Treatment Group

There were no significant or marginally significant changes. There were possibly significant increases in ornithine and sarcosine, and possibly significant decreases in hydroxyproline, urea, and "homocystine + homocysteine" (note that due to measurement methods this is a total of homocystine and homocysteine).

### Placebo Group

There were no significant or marginally significant changes. There was a possibly significant increase in sarcosine and taurine, and a possibly significant decrease in 1-methyl-histidine, hydroxy proline, methionine sulfoxide, and phosphoserine.

### Behavioral Results

#### Effect of Supplement on Symptoms

One of the original hypothesis is "Will the treatment group improve more than the placebo group on one or more of the measures of autism severity?" The results for the assessment tools are shown in Table 10. For the PGI-R Average Change, the supplement group had a significantly greater improvement than the placebo group (0.67 +/- 0.65 vs. 0.34 +/- 0.54, p = 0.003). The other three assessments had slightly greater improvements in the supplement group than in the placebo group, but none of the differences were statistically significant. (Since the analysis included four comparisons, we define significant as p = 0.05/4 = 0.01).

**Table 10:** Summary results for the four autism assessment tools, for the Arizona and National groups combined.

	Placebo-Pre	Placebo-Post	Difference	Supplement- Pre	Supplement Post	Difference	P-Value
**PGI-R (Average Change)**			**+0.34** **+/- 0.54**			**+0.67** **+/- 0.65**	**0.008**

ATEC (Total)	59 +/- 28	50 +/- 27	-9.6	63 +/- 22	51 +/- 20	-11.9	n.s.

SAS	5.2 +/- 2.6	5.2 +/- 2.6	0.0	5.4 +/- 2.0	5.1 +/- 2.2	-0.3	n.s.

PDD-BI (Autism Composite)	-66 +/- 64	-79 +/- 68	-13.4	-62 +/- 51	-78 +/- 52	-16.4	n.s.

Note that the ATEC, SAS and PDD-BI are assessed at the beginning and end of the study, whereas the PGI-R is only assessed at the end, as it only evaluates the changes observed. Only the PGI-R found a significant difference between the supplement and placebo groups, so it is highlighted in a bolder font.

**Figure 6:**
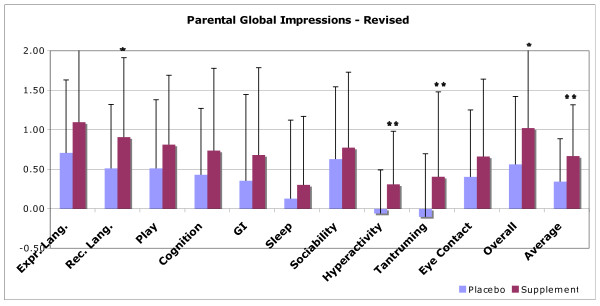
**Results for the Parent Global Impressions - Revised, for the Arizona and National groups combined**. The supplement group had greater improvements in the PGI-R Average score (p = 0.008), and in the subscores for Receptive Language (p = 0.03), Hyperactivity (p = 0.003), Tantrumming (p = 0.009), and Overall (p = 0.02). The y-axis is from -3 (much worse) to 0 (no change) to +1 (slightly better) to +2 (better) to +3 (much better).

Because the results for the PGI-R were significant, the detailed results of the PGI-R are displayed in Figure 6 and Table 11. There are a total of 11 comparisons, so in this section we will define "significant" as p < 0.005, "marginally significant" as p < 0.01, and "possibly significant" as p < 0.05. Overall, the supplement group had a significantly greater improvement in Hyperactivity (p = 0.003), a marginally significant greater improvement in Tantrumming (p = 0.009), and possibly significant greater improvements in Receptive Language (p = 0.03), and Overall (p = 0.02). There are possible trends (p < 0.10) towards improvement in the areas of Expressive Language (p = 0.06) and Play (p = 0.09)). The other areas of the PGI-R yielded non-significant differences between the treatment and the placebo group, but the treatment group consistently scored higher in those other areas, suggesting that larger studies may be needed to investigate possible differences in those other areas.

**Table 11:** Detailed results for the Parent Global Impressions-Revised, for the Arizona and National groups comibined.

	Placebo Group (n = 51)	Supplement Group (n = 53)	Difference	P-Value
Expr. Lang.	0.71 +/- 0.92	1.09 +/- 1.11	0.39	0.06

**Rec. Lang**.	**0.51** **+/- 0.81**	**0.91** **+/- 1.01**	**0.40**	**0.03**

Play	0.51 +/- 0.87	0.81 +/- 0.88	0.30	0.09

Cognition	0.43 +/- 0.84	0.74 +/- 1.04	0.31	n.s.

GI	0.35 +/- 1.09	0.68 +/- 1.11	0.33	n.s.

Sleep	0.13 +/- 0.99	0.30 +/- 0.87	0.17	n.s.

Sociability	0.63 +/- 0.92	0.77 +/- 1.00	0.15	n.s.

**Hyperactivity**	**-0.06** **+/- 0.55**	**0.31** **+/- 0.67**	**0.37**	**0.003**

**Tantrumming**	**-0.10** **+/- 0.80**	**0.40** **+/- 1.08**	**0.51**	**0.009**

Eye Contact	0.40 +/- 0.85	0.66 +/- 0.98	0.26	n.s.

**Overall**	**0.56** **+/- 0.86**	**1.02** **+/- 1.00**	**0.46**	**0.02**

**Average** **(of all the other scores)**	**0.34** **+/- 0.54**	**0.67** **+/- 0.65**	**0.32**	**0.008**

Results with p-values below 0.05 are highlighted in a larger, bold font.

#### Medication Effects

Since some participants were taking psychotropic medications, a comparison of the PGI-R scores was made between the treatment group taking and not taking psychotropic medications. There were no significant differences, but there was a trend that the group taking psychotropic medications had less improvement than those not taking medications for three subscales of the PGI-R: expressive language (-46%, p = 0.08), play (-47%, p = 0.09), sociability (-52%, p = 0.09).

#### Age Effects

**Figure 7:**
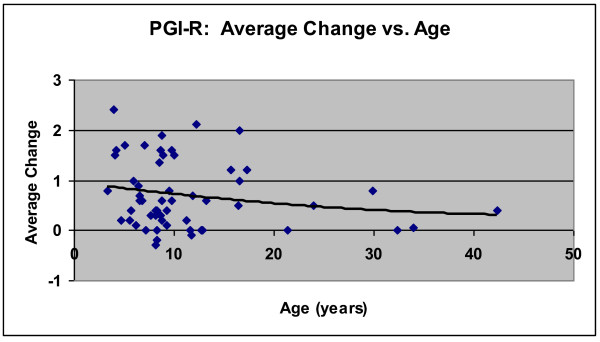
**Average Change on the PGI-R vs. Age, for the Arizona and National Treatment group**. There is a slight, non-significant correlation of the Average Change vs. age, as indicated by the trendline. The y-axis is from -3 (much worse) to 0 (no change) to +1 (slightly better) to +2 (better) to +3 (much better).

The correlation of the Average Change of the PGI-R vs. age was calculated for the treatment group, and found to be r = - 0.20 (not significant). Figure 7 shows the Average Change of the PGI-R vs. Age. Below age 20 there are many Average Changes above 1, but after age 20 there are no Average Changes above 1, but some are still positive. So, the greatest benefit appears to be for people under the age of 20, but there were some reported improvements for people up to the highest age in the study (mid-forties).

Figure 7 also provides a visual of the large variation in response; some participants reported little or no improvement, some reported moderate improvement (1 = slightly better), and some reported substantial improvement (2 = better, 3 = much better).

There were only 3 female participants in the supplement group, which is too few to make any generalization - their Average Change scores were 0.2, 0.5, and 0.8, similar to those of the males.

#### Correlation with Biomarkers

The correlation of the Average Change of the PGI-R vs. initial (baseline) biomarkers of nutritional status were calculated for the supplement group of the Arizona study (biomarkers were only measured for the Arizona group). The biomarkers with correlations larger in magnitude than 0.46 (corresponding to p < 0.05) are listed in Table 12. However, since multiple comparisons were made, none of these correlations were significant after making a Bonferroni correction, so these correlations are at most possibly significant. Vitamin K had the strongest correlation with the Average Change of the PGI-R, followed by biotin and lipoic acid.

**Table 12:** Correlation of PGI-R Average Score with Biomarkers, for the Arizona Treatment group only.

Biomarker	Correlation Coefficient (r)	p-value
Vitamin K	- 0.74	p < 0.01

Biotin	- 0.67	p < 0.01

Lipoic Acid	+ 0.52	p < 0.05

Correlations of 0.46 or larger in magnitude are reported, corresponding to a p-value of 0.05, in order starting with the strongest correlations.

#### Regression Analysis

Since three biomarkers had possibly significant correlations with the Average Change of the PGI-R, a regression analysis was conducted to determine which set of biomarkers were the best predictors of improvement, and hence determine which children were most likely to benefit from the vitamin/mineral supplement. The results are displayed in Table 13. A very strong and highly significant association of the biomarkers with the Average Change of the PGI-R was found (adjusted R2 = 0.61, p < 0.0005), with the most significant biomarkers being vitamin K (p = 0.03) and Biotin (p = 0.04).

**Table 13:** Result of Regression Analysis of PGI-R Average Score with Biomarkers, for the Arizona Treatment Group only

	PGI-R
**Significant Biomarkers**	

Adjusted R^2^	0.61

P-value	0.0005

Primary variables	Vit K (p = 0.03) Biotin (p = 0.04)

## Discussion

### Discussion of Nutritional/Metabolic Changes

In general, although we focus on averages in the following sections, it is important to realize the breadth of the distributions. So, although children with autism may (for example) have average levels of vitamin B1, there is a subset with lower levels, so increases in the average level of vitamin B1 may be more beneficial to those with lower levels. Similarly, although children with autism tend to have lower levels of (for example) glutathione, some children have normal levels, but many have low levels; improvements in the average level are probably most beneficial to those with lower levels.

Also, it is important to point out that "average" levels of the neurotypical group may not necessarily be optimal, as they were on typical western diets that are probably not nutritionally optimal.

### Vitamins

Overall, the supplement increased the level of most vitamins, including vitamins B1, B3, B5, B6, folic acid, B12, C, E, and biotin. It appears that higher levels of vitamin B2 are needed in the supplement to affect blood levels. Carotene levels improved, but were still somewhat low, so higher amounts are needed. It is interesting that supplementation with carotenes and modest amounts of vitamin A did not significantly alter vitamin A levels, which remained normal; this is consistent with the body only converting carotenes to vitamin A if vitamin A levels are low.

The supplement also improved two functional biomarkers in urine, FIGLU and methylmalonic acid, indicating the supplement decreased the need for folic acid and vitamin B12, respectively. Levels of FIGLU and methylmalonic actually decreased somewhat below the levels of the neurotypical controls; this may be a good result, as some typical children do not have optimal nutritional intake. SAM levels normalized, and uridine levels improved but did not normalize, suggesting that more vitamin B12 and/or folinic might be needed.

Vitamin C levels in the autism group were initially somewhat above that of the neurotypical group, and the supplement raised those levels significantly. This is probably beneficial, as the children with autism initially had high oxidative stress, and the supplement significantly decreased the level of oxidative stress, probably in part due to the vitamin C in the supplement.

Vitamin D decreased in both the treatment and placebo group - this was apparently a seasonal effect, as the study began in the summer/fall, and ended in the fall/winter, and most vitamin D in the body is produced by sunlight. It appears that much higher levels of vitamin D are needed to affect blood levels of vitamin D.

### Minerals

Caution needs to be used in interpreting the results for minerals, as absolute levels are not necessarily the best way to measure body stores and the need for minerals. Also, there is some debate over which compartment (WB, RBC, serum, urine, etc) is the best to use in measuring a given mineral. For a full discussion of these complex issues, see Gibson 2005 [32].

Overall, the supplement tended to increase the levels of many essential minerals, including calcium, iodine, lithium, manganese, molybdenum, and selenium. The increase in lithium levels was large (this form of lithium was very well absorbed), so less lithium may be needed in future studies. Magnesium levels in whole blood significantly increased and normalized, but there was a possible decrease in RBC levels, and no change in serum levels, which is somewhat inconsistent; however, overall, it seems that the increase in whole blood levels was the most significant/important.

The supplement also normalized RBC iron, from slightly (but significantly) higher initial levels compared to the neurotypical average, to levels close to that of the neurotypical group. RBC iron is a measure of the total iron in the RBC, and about 65% of the body's iron is in the RBC [33], so RBC iron may be a reasonable indicator of total body stores of iron. The importance of elevations in RBC iron (statistically significant), serum ferritin and serum iron (non-significant) are unclear; supplementation resulted in all three declining to the neurotypical average level. Increases in serum ferritin and altered iron metabolism are known to occur with inflammation. This may also hold for increased oxidative stress. In the baseline evaluations the regression analysis found that RBC iron was significantly associated with all three assessments of autism severity (p < 0.01) [20]. Based on these findings, further evaluation of iron metabolism in autism is warranted.

Levels of copper, and zinc were not significantly affected (note that copper is not included in the supplement since children with autism seem to generally have adequate or slightly high levels of it). It should be pointed out that zinc levels began and ended in the normal range. It is possible that increased zinc supplementation would normalize the slightly elevated copper levels. Chromium decreased to a normal level, but the change was not significant.

Regarding the placebo group, there was a significant increase in manganese, but other changes appeared to be small fluctuations around the average level in neurotypical children.

### Sulfation

The supplement substantially improved sulfate status, but sulfate levels were still low, suggesting that higher levels of MSM or other sources of sulfate such as Epsom salt (magnesium sulfate) baths are needed. Sulfur is the third most common mineral in the body [34]. Most sulfate is produced *in vivo *by metabolism of cysteine [35]. Sulfation is important for many reactions including detoxification, inactivation of catecholamines, synthesis of brain tissue, sulfation of mucin proteins which line the gastrointestinal tract, and more. The measurement of total plasma sulfate involves many substances in the plasma, including neurotransmitters, steroids, glycosaminoglycans, phenols, amino acids, peptides, and other molecules. Low free and total plasma sulfate in children with autism has been previously reported in two studies [36,37], and is consistent with four studies [36,38-40] which found that children with ASD had a significantly decreased sulfation capacity compared to controls, based on decreased ability to detoxify paracetamol (acetaminophen). The finding of low plasma sulfate is also consistent with a large study that found high sulfate in the urine of children with autism [41], as sulfate wasting in the urine partly explains low levels in the plasma. ATP is required for the kidneys to resorb sulfate, and the accompanying study [20] found that ATP was moderately correlated with levels of free and total plasma sulfate (r = 0.32 and 0.44, respectively), so this suggests that low levels of ATP are a contributor to decreased sulfate in children with autism. One study [41] also reported high levels of urinary sulfite in children with autism, suggesting that there was a problem of converting sulfite to sulfate in the mitochondria. In 38% of cases (14/38) urinary sulfite and sulfate levels improved by giving 50 mcg of molybdenum, presumably since the enzyme for converting sulfite to sulfate (sulfite oxidase) contains molybdenum. The vitamin/mineral supplement in this study contained molybdenum (150 mcg for a 30 kg child), so this may also have contributed to increases in sulfate levels.

### Methylation, Glutathione and Oxidative Stress

**Figure 8:**
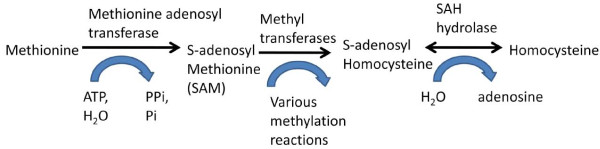
**Conversion of Methionine to SAM to SAH to Homocysteine**. Homocysteine is then either recycled to methionine or converted into cystathionine.

Methylation improved to near-normal levels, as indicated by improvements in SAM and uridine. SAM is the primary methyl donor for methylation of DNA, RNA, proteins, phospholipids, and neurotransmitters. The improvement in SAM may in part be due to improvements in ATP, since that is the co-factor needed to convert methionine to SAM. The methylation pathway is diagrammed in Figure 8.

**Figure 9:**
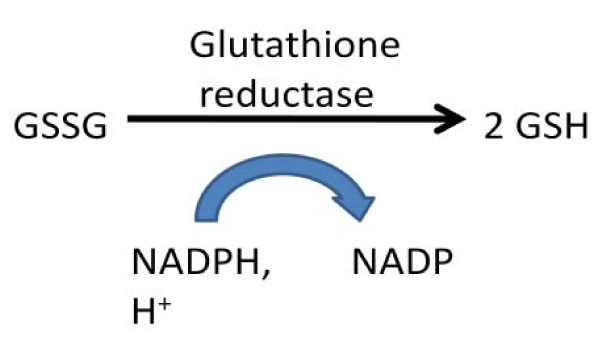
**Reduction of GSSG to GSH (net result of a more complex process which involves FADH)**.

The supplement also substantially improved glutathione (an important anti-oxidant and defense against toxic metals). The supplement substantially reduced oxidative stress to near-normal levels, as evidenced by improved ratio of GSSG:GSH and improved levels of nitrotyrosine. NADPH is the co-factor needed to recycle GSSG to GSH (see Figure 9), so normalizing the level of NADPH probably was the major factor in improving the GSSG:GSH ratio.

Previous studies [9,10] have demonstrated that oral folinic acid, oral trimethylglycine, and subcutaneous injections of methyl Vitamin B12 were able to greatly improve methylation, glutathione, and oxidative stress, similar to the results here. This suggests that the oral supplement used in this study may be a reasonable alternative to subcutaneous injections of methyl-B12. Oral intake of vitamin B12 has a complex absorption mechanism involving "intrinsic factor", and typically only 1% of oral vitamin B12 is absorbed, so it is interesting that the levels used in this study were sufficient to substantially improve methylation, glutathione, and oxidative stress. The vitamin C in the present supplement probably also helped reduce oxidative stress.

The current observed improvements in methylation and GSH are similar to effects of treatment with NADH [42] and ribose [42], but neither ribose nor NADH had significant effect on improving levels of GSSG after two weeks.

### ATP, NADH, NADPH, CoQ10

ATP, NADH, NADPH, and CoQ10 are important co-factors for many metabolic processes in the body. ATP is a primary energy source for the body and the brain. The CoQ10 in the supplement was very well absorbed, so that the relatively modest dosage resulted in a large, significant increase in CoQ10 levels. The supplement significantly increased the plasma levels of ATP, NADH, and NADPH, from about 25% below normal to normal levels. Plasma ATP may be a biomarker of general ATP status in the body, and may be related to overall level of ATP, and/or the ability to recycle ATP, and/or the ability to transport ATP where needed - more research is needed to interpret the importance of plasma ATP. Many children with autism have low muscle tone and impaired endurance, and it is interesting to hypothesize if those symptoms relate to decreased ATP levels, and if improvements in plasma ATP will result in improvements in muscle tone and endurance - those symptoms were not assessed in this study, but would be interesting to assess in future.

The results of vitamin/mineral supplementation on ATP, NADH, NADPH is similar to the results of supplementation with NADH [42] and ribose [42], since NADH is easily converted to NADPH, which is a co-factor for making ribose, which is a building block of ATP, NADH, NADPH, and many other important substances.

### Primary and Secondary Amino Acids

There were no significant or marginally significant changes. Most small changes in primary and secondary essential amino acids appeared to involve modest fluctuations around the average level of the neurotypicals. One possible exception is the slight decrease in serine coupled due to increase in glycine. Serine is converted to glycine in an enzymatic reaction requiring tetrahydrofolate as a co-factor - this may suggest a small increase in production of tetrahydrofolate.

### Medication Effects

A previous paper [20] reports on a comparison of the nutritional and metabolic status of the participants taking medications vs. those not taking medications. The only differences with a p-value less then 0.01 were lower RBC copper (-9% lower, p = 0.001) and higher plasma methionine sulfoxide (+35% higher, p = 0.002) for the autism medication group compared to the autism no-medication group. The sample size in this paper is too small to determine if medications had an effect on changes in the nutritional and metabolic status of the treatment group, but since no changes in medication were made during the study, this was probably a minor effect at most.

### Discussion of Placebo

The placebo group had a few significant changes, including significant increases in biotin, CoQ10, WB manganese, and SAM. In all four cases the supplement group had similar changes (biotin, WB manganese) or larger changes (CoQ10, SAM). Some of these findings might be due to laboratory error (drift in standards), but that seems unlikely. Some of the changes may be due to random fluctuations in diet, or possibly due to seasonal effects (i.e., baseline values were measured in summer/fall (June-October) and final values in fall/winter (September to January). Finally, it may be that the natural plant-based flavorings used in the placebo (not in the supplement) contained modest amounts of biotin and other nutrients.

### Discussion of Effect on Symptoms

The supplement group had significantly greater improvement that the placebo group on the Average Change of the PGI-R. The supplement group had greater improvement than the placebo group on all of the subscales, with several of the results being significant (p < 0.005), marginally significant (p < 0.01), or possibly significant (p < 0.05). Although the magnitude of the effects were modest, the supplement group reported roughly twice the improvement as did the placebo group on the Average Change score (the average of all the PGI-R scores). Since the supplement resulted in many significant improvements in nutritional and metabolic status after three months, we hypothesize be that the child's overall health and learning ability is improved at that point, but that more time may be needed for the increase in learning ability to fully translate into greater skills in language, social understanding, and behavior.

For the other three assessment tools, the supplement group also had a slightly greater improvement than did the placebo group, but the effect was not significant. This suggests that the PGI-R is more sensitive at detecting changes, which is what it was designed for, whereas the other scales measure overall autism severity. It should be noted that the PGI-R uses a 7-point scale, whereas the ATEC and PDD-BI use a 3 to 4 point scale, and that may be part of the reason why the PGI-R appears to be more sensitive. More importantly, the PGI-R directly assesses the degree of improvement, whereas the other assessment tools only indirectly assess the degree of improvement by calculating a small difference between two large numbers (initial and final), which leads to a greater uncertainty in the degree of improvement.

#### Correlation with Biomarkers

The correlation of three biomarkers with the Average Change of the PGI-R is interesting. The correlations need to be interpreted cautiously, because the sample size (only the Arizona treatment group) is small. The autism group had lower levels of biotin than did the neurotypical group (-20%, p = 0.001) at the start of the study, and the supplement significantly increased levels of biotin (+51%, p = 0.008) in the treatment group. So, it makes sense that children with low levels of biotin would be more likely to benefit from supplementation. Biotin is an important co-factor for four carboxylases that regulate gluconeogenesis (generation of glucose from non-carbohydrate sources), fatty acid synthesis, and the Krebs cycle.

The autism group initially had levels of vitamin K that were similar to the control group. Vitamin K was the only vitamin not included in the supplement, and the level did not significantly change (+15%, n.s.) during the study. The primary role of vitamin K is in blood coagulation, which is not reported as a common problem in autism, which is why it was not included in the supplement in this study. However, a previous study [20] found by regression analysis that levels of vitamin K were somewhat associated with variation in the severity of autism. So, the correlation of vitamin K with degree of improvement is puzzling.

What biotin and vitamin K have in common is that both are made in substantial amounts by beneficial intestinal bacteria. It is estimated that approximately half of the biotin and half of the vitamin K in humans comes from their intestinal bacteria [43]. One of the common causes of biotin or vitamin K deficiency is antibiotic usage, because some antibiotics destroy the beneficial bacteria that produce them [43]. Several studies have reported that one major difference in the medical history of children with autism compared to neurotypical children is a much higher usage of oral antibiotics antibiotic [44-47]. So, it could be that excessive oral antibiotic usage contributed to lower levels of biotin and vitamin K in some children. Vitamin K occurs in two natural forms, vitamin K1 (phylloquinone) produced by plants, and vitamin K2 (menaquinone) produced by intestinal bacteria. In this study we measured total vitamin K (K1 plus K2); in future studies it would be interesting to measure both forms individually.

We analyzed the possible correlation of levels of vitamin K and biotin in the autism group at the start of the study, and found that they were significantly correlated (r = 0.44, p < 0.001). This is consistent with both being partially produced by beneficial intestinal bacteria. One study found a very high correlation of GI problems with autism severity (r = 0.59, p < 0.001) [48]. So, it appears that the correlation of improvement in autism symptoms with biotin and vitamin K may relate to a lack of beneficial bacteria which produce biotin and vitamin K, so that supplementation with biotin was beneficial. This suggests that supplementation with vitamin K would be beneficial, especially for those with low levels of vitamin K (the standard deviation of vitamin K levels in the autism group was large).

The negative correlation of lipoic acid with the Average Change of the PGI-R is interesting. The supplement did not contain lipoic acid, and it did not affect levels of lipoic acid, so it appears that children were more likely to improve if they already had sufficient lipoic acid, whereas a lower level of lipoic acid seemed to be associated with less improvement. However, this correlation is not as strong as that for biotin and vitamin K. More research into supplementation with lipoic acid may be warranted.

#### Regression Analysis

The regression analysis found that the Average Change of the PGI-R was very strongly associated with several biomarkers, with vitamin K and biotin being the most significant. This suggests that children with low biotin or low vitamin K were most likely to benefit from the multi-vitamin/mineral supplement, for reasons discussed in the preceeding section. This suggests that vitamin K should be added to future formulations.

It is important to realize that vitamin levels are not independent variables, but are usually significantly correlated with one another, because they often occur in the same foods. So, in interpreting these results, it may be that biotin and vitamin K are also markers of overall nutritional status, and their individual importance may be less important.

#### General Discussion

At the start of the study the children with autism had many statistically significant differences (p < 0.001) in their nutritional and metabolic status compared to the neurotypical group [20], including: Low levels of biotin, glutathione, SAM, plasma ATP, NADH, NADPH, plasma sulfate (free and total), and plasma tryptophan; also high levels of oxidative stress biomarkers and evidence of impaired methylation (high uridine). By the end of the treatment study, these biomarkers had all improved or even normalized. Also, the baseline study [20] found that levels of several vitamins, minerals, and amino acids were strongly associated with variation in autism severity. Vitamins and minerals act as enzymatic co-factors for hundreds of important enzymatic reactions in the body, so low levels of them can result in impaired metabolic functioning. Also, many genetic variations result in impaired enzymatic activity, resulting in an increased need for vitamin/mineral co-factors for normal metabolic functioning. This study was only able to assess a limited portion of human metabolism, and it is likely that other metabolic problems exist in children with autism and possible that the vitamin/mineral supplement could improve other problems as well as those reported here. For example, vitamins and minerals are required co-factors for the production of many neurotransmitters and their pre-cursors, so vitamin/mineral supplementation may have also affected neurotransmitter status, and that may have contributed to improvements in autism severity and overall symptoms. So, it is not surprising that nutritional supplementation would improve metabolic functioning in some children with autism, and it is very interesting that nutritional supplementation also resulted in significant improvements in the Average Score of the PGI-R, as well as improvements in several of its subscores. Some children improved much more than others, presumably because some had a greater need for nutritional supplementation.

This study is consistent with several other studies that reported that vitamin/mineral supplementation is beneficial in treating children with autism. A 30-week, double-blind, placebo-controlled study [12] of high-dose vitamin C (110 mg/ kg) found a reduction in autism severity. One open-label study [49] found that micronutrient supplementation was comparable or more effective than treatment with pharmaceuticals in terms of improvements in the Childhood Autism Rating Scale, Childhood Psychiatric Rating Scale, Clinical Global Impressions, and Self-Injurious Behavior. A small randomized, double-blind, placebo-controlled study of a moderate-dose vitamin/mineral supplement was found to be beneficial to children with autism, primarily in the areas of sleep and gastrointestinal symptoms [14]. There have been many studies of high dose vitamin B6 therapy in children with autism [13], with most showing beneficial effects; those studies investigated very high dosages, generally 500-1000 mg, compared to the current study which investigated a lower dosage (40 mg for a 30 kg child) which is still substantially higher than the RDA (0.6 mg), and was sufficient to substantially increase P5P levels inside RBC (+187%, p < 0.001). Some children and adults may benefit from adding high-dosage vitamin B6 to a broad-spectrum vitamin/mineral supplement such as investigated in this study.

Compared to other treatments, the administration of a vitamin/mineral supplement requires only a few minutes a day, is relatively inexpensive, and is very safe. Although it will not help all children and adults with autism, it appears that a significant percentage are likely to improve to some degree after only three months, and longer-term use is likely very safe and may result in even greater benefits. Also, the vitamin/mineral supplement improved many nutritional and metabolic problems. So, vitamin/mineral therapy seems to be a reasonable adjunct therapy for helping some children and adults with autism, and can be easily used in conjunction with other therapies (behavior therapy, speech therapy, etc.).

### Limitations of this study

1) The diagnosis of an autism spectrum disorder by a qualified medical professional was verified in writing, but there no additional verification. Similarly, for the neurotypical children, no additional verification was made beyond the parental report. The supplement group included a somewhat higher fraction of individuals with classic autism than did the placebo group, since random assignment was done and severity of diagnosis was not controlled for. However, the effect on the results is probably small, since the analysis investigated the change in symptoms, not the final symptoms only.

2) The sample size was large enough to observe many major significant differences between the two groups; but a larger sample size is needed for appropriate statistical power for more subtle, possibly significant differences.

3) The formulation of the supplement was very good; the present data suggests ways to further improve composition and dosage optimization and titration.

4) Seasonal changes slightly affected some results (vitamin D) and possibly others.

5) The placebo contained small amounts of natural plant-based extracts that may have slightly affected some results.

6) Some of the children (45%) were taking various types of medications, which did not change during the study. A comparison of the baseline levels of the autism groups taking and not-taking medications revealed little difference between the two groups in their nutritional and metabolic status [20]. There was a trend that the medicated group had less improvement than the unmedicated group in the Average Score of the PGI-R.

7) The length of the study (three months) may not have been long enough to observe the full-effect of the supplement, and longer treatment may result in larger effect.

## Conclusions

The vitamin/mineral supplement was found to be generally well-absorbed and metabolically active, resulting in improvements in biotin, glutathione, methylation, oxidative stress, ATP, NADPH, NADPH, and sulfate. The supplement was well-tolerated, with few side-effects, although for a few participants their individually titrated dose was lower than originally prescribed.

The supplement group improved significantly more than the placebo group on the PGI-R Average Change and on several of the PGI-R subscales. On the PGI-R subscales, the most significant improvements were (in order) in the areas of Hyperactivity, Tantrumming, Overall, and Receptive Language. We hypothesize that longer treatment may result in greater improvements. There was wide variation in degree of improvement, with some participants experiencing little benefit, and some experiencing moderate or substantial benefit.

The data from this study strongly suggests that oral vitamin/mineral supplementation is beneficial in improving the nutritional and metabolic status of children with autism, and in reducing their symptoms. Based on the present findings, vitamin/mineral supplementation should be considered as an adjunct therapy for most children and adults with autism, especially when any of the metabolic problems discussed in this paper are documented as present.

The data from this study serves as a useful guide for future formulations of vitamin/mineral supplements for children with autism. Additional sources of sulfate, such as MSM or Epsom salt baths, may be needed to normalize sulfate levels.

## Competing interests

The vitamin/mineral formulation designed for this study by JBA and TA has since been improved and made available as an over-the-counter product, Syndion, by Yasoo Health, but none of the authors receive any royalties from the sale of Syndion or any Yasoo Health products.

JBA receives free supplements from Yasoo Health for personal use. Yasoo Health is one of many companies which sponsor the annual Zoowalk for Autism Research in Phoenix, Arizona, which partially funds some of his autism research. JBA is the President of Autism Conference of America, and Yasoo Health is one of many companies which sometimes exhibit at their autism conferences.

TA is an employee of Health Diagnostics and Research Institute (formerly Vitamin Diagnostics), a CLIA-approved medical laboratory which conducted many of the medical tests for this study.

DQ is an employee of Doctor's Data, a CLIA-approved medical laboratory which conducted many of the medical tests in this study.

The other authors do not have any competing interests to declare.

## Authors' contributions

JBA was the principal investigator, oversaw the study design, conducted most of the data analysis, and wrote most of the paper. TA oversaw the laboratory measurements at Health Diagnostics, and assisted with interpreting the results and editing the paper. SMM was the study physician, oversaw patient care, and assisted with interpreting the results and editing the paper. RAR assisted with statistical analysis and editing the paper. DQ oversaw the laboratory measurements at Doctors Data, and assisted with interpreting the results and editing the paper. EG was the lead study nurse, and supervised many of the study participants. EG was the study coordinator, and assisted with participant recruitment and study oversight. ML, JM, SA, and SB were study nurses and supervised study participants. WL assisted with data entry and analysis. All authors read and approved the final version of the paper.

## Pre-publication history

The pre-publication history for this paper can be accessed here:

http://www.biomedcentral.com/1471-2431/11/111/prepub
